# Galectin-1 promotes gastric cancer peritoneal metastasis through peritoneal fibrosis

**DOI:** 10.1186/s12885-023-11047-2

**Published:** 2023-06-17

**Authors:** Xianhe Shen, Huilan Liu, Haihua Zhou, Zhiyi Cheng, Guiyuan Liu, Chuanjiang Huang, Rongrong Dou, Fuxing Liu, Xiaolan You

**Affiliations:** 1 Department of Gastrointestinal Surgery, The Affiliated Taizhou People’s Hospital of Nanjing Medical University, Taizhou, 225300 Jiangsu China; 2 Oncology department, The Affiliated Taizhou People’s Hospital of Nanjing Medical University, Taizhou, 225300 Jiangsu China; 3 Department of the Pathology, The Affiliated Taizhou People’s Hospital of Nanjing Medical University, Taizhou, 225300 Jiangsu China

**Keywords:** Gastric cancer, Peritoneal metastasis, Galectin-1, Cancer-associated fibroblast, Peritoneal fibrosis

## Abstract

**Background:**

Peritoneal metastasis is one of the main causes of death in patients with gastric cancer (GC). Galectin-1 regulates various undesirable biological behaviors in GC and may be key in GC peritoneal metastasis.

**Methods:**

In this study, we elucidated the regulatory role of galectin-1 in GC cell peritoneal metastasis. GC and peritoneal tissues underwent hematoxylin–eosin (HE), immunohistochemical (IHC), and Masson trichrome staining to analyze the difference in galectin-1 expression and peritoneal collagen deposition in different GC clinical stages. The regulatory role of galectin-1 in GC cell adhesion to mesenchymal cells and in collagen expression was determined using HMrSV5 human peritoneal mesothelial cells (HPMCs). Collagen and corresponding mRNA expression were detected with western blotting and reverse transcription PCR, respectively. The promoting effect of galectin-1 on GC peritoneal metastasis was verified in vivo. Collagen deposition and collagen I, collagen III, and fibronectin 1 (FN1) expression in the peritoneum of the animal models were detected by Masson trichrome and IHC staining.

**Results:**

Galectin-1 and collagen deposition in the peritoneal tissues was correlated with GC clinical staging and were positively correlated. Galectin-1 enhanced the ability of GC cells to adhere to the HMrSV5 cells by promoting collagen I, collagen III, and FN1 expression. The in vivo experiments confirmed that galectin-1 promoted GC peritoneal metastasis by promoting peritoneal collagen deposition.

**Conclusion:**

Galectin-1-induced peritoneal fibrosis may create a favorable environment for GC cell peritoneal metastasis.

**Supplementary Information:**

The online version contains supplementary material available at 10.1186/s12885-023-11047-2.

## Background

Gastric cancer (GC) is one of the most common malignant tumors of the digestive tract. Worldwide, GC morbidity and mortality are ranked fifth and third, respectively [1, 2]. Early-stage GC is asymptomatic, where approximately 80–90% of patients with GC are diagnosed with metastasis in the late stage [3]. The peritoneum is the most frequent GC metastatic site: approximately 14% of patients with GC develop peritoneal metastasis upon their GC diagnosis [4], and peritoneal metastasis is the most frequent mode of metastasis after radical gastrectomy. The lack of effective treatment contributes to GC-related mortality and is a cause of postoperative mortality in patients with GC. To date, the mechanism of GC peritoneal metastasis has not been determined. Exploring the mechanism of GC peritoneal metastasis would provide new ideas for treating peritoneal metastasis of GC.

As early as 1889, Paget proposed the seed and soil theory based on the study of breast cancer metastasis [5], and the theory has been widely accepted as the classical theory of GC peritoneal metastasis [6]. The seed and soil theory states that GC peritoneal metastasis is not only determined by GC cells (seeds), but also by the local conditions of the peritoneum (soil), which are crucial in tumor metastasis. In short, tumor metastasis involves interaction between the primary tumor and the local microenvironment of the new metastatic site. Therefore, by providing various cytokines that promote tumor invasion and metastasis, tumors promote peritoneal changes to facilitate the adhesion of shed tumor cells to the peritoneum, which is an important basis for the occurrence of GC peritoneal metastasis [6, 7]. This process involves interaction between the primary tumor and peritoneal mesenchymal cells and the extracellular matrix (ECM) [8].

A tumor is composed of tumor cells and the tumor microenvironment (TME) comprising tumor cells and diverse stromal cells, chemokines, and cytokines, which facilitate tumor growth and metastasis [9, 10]. It was recently reported that the TME is key in tumor development and progression [10]. The TME refers to the cellular environment in which tumors or cancer stem cells exist and is a complex functional environment that contains ECM and a variety of stromal cells, such as mesenchymal stem cells, immune cells, and fibroblasts [11]. Fibroblasts can be activated into cancer-associated fibroblasts (CAFs) in the early stage of tumorigenesis. CAFs are the most abundant cell type in all cancers and promote tumor cell migration and alter epithelial tumor cell metabolism [12, 13]. Given their superior numbers and strong crosstalk as compared to tumor cells, CAFs are particularly important in the TME and in regulating tumor occurrence and development. CAFs promote GC cell growth, invasion, and metastasis by secreting cytokines [14, 15].

GC CAFs highly express galectin-1 (Gal-1, encoded by the *LGALS1* gene), which regulates tumor cell malignant biological behaviors [16, 17]. Galectin-1 is one of the 15 members of the β-galactose-binding protein family known as galectins and is a homodimer protein composed of 14.5-kDa subunits that maintain morphological stability through hydrophobic bond interaction at the monomeric interface [18]. Galectin-1 is overexpressed in numerous malignant tumors, including GC [17]. Exogenous galectin-1 inhibited tumor cell–ECM adhesion and promoted tumor cell shedding through competitive binding with matrix glycoprotein or cell surface glycocomplex [19]. Meanwhile, laminin in the basement membrane, fibrin, and other glycoproteins provide the necessary epitopes for galectin-1 to cross-link with ECM and encourage galectin-1 to mediate tumor cell adhesion to the ECM. Furthermore, galectin-1 promotes homotype cell aggregation and invasion and subsequently angiogenesis. The quadruple action of galectin-1 satisfies the four steps in GC peritoneal metastasis: cancer cells escape from the primary tumor, accumulate in the abdominal cavity to resist anoikis, adhere to distant peritoneum and invade it, and proliferate through neovascularization. Therefore, we hypothesized that galectin-1 is crucial in GC peritoneal metastasis.

However, the relationship between galectin-1 and GC peritoneal metastasis is limited to theoretical inference, and the specific mechanism is not clear. Exploring the molecular mechanism of galectin-1 promotion of GC invasion and metastasis will provide new molecular markers for early diagnosis and prognostic evaluation for treating GC and improve the clinical diagnosis and treatment of GC peritoneal metastasis, which would be greatly important for improving patients’ survival rates.

## Methods

### Tissue samples

The GC tissues and matched peritoneal tissues were from patients who had undergone radical gastrectomy at the Taizhou People’s Hospital Affiliated to Nanjing Medical University Department of Gastrointestinal Surgery from October 2021 to April 2022. None of the patients had received preoperative neoadjuvant chemoradiotherapy. The Taizhou People’s Hospital Ethics Committee approved the study and all patients signed informed consent forms to participate in the clinical study prior to surgery. The patients’ clinical tumor-node-metastasis (TNM) staging was performed with reference to the eighth edition American Joint Committee on Cancer (AJCC) staging system. The peritoneal tissues were obtained from the left parietal peritoneum through the upper abdominal incision, measuring approximately 1.0*2.0 cm in size. The tissue specimens were fixed in 10% formalin, paraffin-embedded, sectioned to 4-μm thickness, and stained with hematoxylin–eosin (HE) and Masson trichrome. The submesothelial ECM of the stained tissues was determined, where three independent measurements were calculated for each tissue section, then all data were summarized.

### Reagents and antibodies

The antibodies against galectin-1(ab138513),and GAPDH(ab9485) were from Abcam (Cambridge, UK). The polyclonal antibodies against collagen I (bs-0578R), collagen III (bs-0549R), and fibronectin 1 (FN1)/Ugl-Y3 (bs-13455R) were from Bioss (Beijing, China). The goat anti-rabbit IgG (sc-2357) and horseradish peroxidase (HRP)-conjugated goat anti-mouse IgG (sc-516102) were from Santa Cruz Biotechnology (Santa Cruz, CA, USA). The MTT assay kit and dimethyl sulfoxide were from Sigma-Aldrich (St. Louis, MO, USA). The Modified Masson’s Trichrome Stain Kit was from Beijing Solarbio Science & Technology Co. Ltd. (G1346; Beijing, China).

### Cell line and culture

The HMrSV5 human peritoneal mesothelial cell (HPMC) line was from BeNa Culture Collection (Beijing, China) and was cultivated in 90% Dulbecco’s modified Eagle’s medium supplemented with 10% (V/V) fetal bovine serum (FBS; Thermo Fisher Scientific, Waltham, MA, USA). An undifferentiated human gastric carcinoma cell line (HGC-27) and a moderately differentiated human gastric adenocarcinoma cell line (SGC-7901) were obtained from Beyotime Biotech Inc. (Shanghai, China) and cultured in RPMI 1640 medium (Thermo Fisher Scientific) containing 100 U/mL penicillin and streptomycin (Gibco, Grand Island, NY, USA) and 10% (V/V) FBS. All cells were cultured at 37 °C in a humidified atmosphere containing 5% (V/V) CO_2_. The cells were passaged by 2.5% trypsinization when they were 80% confluent.

### Lentiviral transduction

The lentiviral vectors carrying a puromycin resistance gene and green fluorescent protein (GFP) for *LGALS1* overexpression were constructed by Genechem Co. Ltd. (Shanghai, China). The negative control (NC) lentiviral vector also carried a puromycin resistance gene and GFP. One day before lentiviral transduction, we seeded 5 × 10^4^ cells per well SGC-7901 or HGC-27 cells on 6-well plates. The amount of virus vector was calculated according to the virus vector titer and the multiplicity of infection of cells in 10 μg/mL polybrene (Sigma-Aldrich). The culture medium was replaced with fresh medium after incubation at 37℃ with 5% CO_2_ for 12 h. After 48-h incubation, 2 µg/mL puromycin (Sigma-Aldrich) was added to the medium to select stably transduced cell lines. Then, the stable transductants were cultured in medium containing 0.5 μg/mL puromycin. Transduction efficiencies were evaluated 72 h after transduction.

### Semi-quantitative reverse transcription PCR (RT-PCR)

HPMCs total RNA was extracted with a RNeasy Mini Kit (Invitrogen, Waltham, MA, USA) for real-time qRT-PCR. First-strand complementary DNA was synthesized by a RT kit (Takara, Shiga, Japan), then the qRT-PCR was performed with an iQ5 Multicolor Real-Time PCR Detection System (Bio-Rad, Hercules, CA, USA) and using SYBR Green (Roche Diagnostics, Mannheim, Germany). The relative expression levels were calculated based on the glyceraldehyde-3-phosphate dehydrogenase (*GAPDH*) gene, where the relative expression was calculated with the comparative threshold cycle method (2^-ΔΔCt^). All experiments were performed in triplicate. Table 1 lists the primers for *LGALS1*, *COL1A1* (collagen I), *COL3A1* (collagen III), *FN1*, and *GAPDH*.

**Table 1:** Primers used for qRT‐PCR in the presentstudy

Gene	Gene Primer sequence (5′ to 3′)
*LGALS1*	F: GCCAGATGGATACGAATTCAAG
	R: GCCACACATTTGATCTTGAAGT
*COL1A1*	F: AAAGATGGACTCAACGGTCTC
	R: CATCGTGAGCCTTCTCTTGAG
*COL3A1*	F: TGAAGGGCAGGGAACAACTTGATG
	R: GGATGAAGCAGAGCGAGAAGTAGC
*FN1*	F: AATAGATGCAACGATCAGGACA
	R: GCAGGTTTCCTCGATTATCCTT
*GAPDH*	F: CCAGCAAGAGCACAAGAGGAAGAG
	R: GGTCTACATGGCAACTGTGAGGAG

### Protein extraction and western blotting

The HPMCs proteins were separated with sodium dodecyl sulfate–polyacrylamide gel electrophoresis and the gel plates were cut prior to hybridisation with antibodies according to the molecular weight of the proteins, then transferred onto nitrocellulose membranes (GE Healthcare Life Sciences, Pittsburgh, PA, USA). The blots were probed with primary antibodies against collagen I, collagen III, FN1, and GAPDH (1:2000 dilution). The secondary HRP-conjugated antibody was also used at 1:2000 dilution. The protein bands were visualized with West Pico Chemiluminescent Substrate (Pierce, Carlsbad, CA, USA) and quantified by densitometric image analysis software (ImageMaster VDS; Pharmacia Biotech, CA, USA). All assays were performed in triplicate.

### Histological examination and immunohistochemistry evaluation

Proteins in human primary GC tissues and animal model peritoneal metastasis tissues were detected with immunohistochemistry (IHC) according to our previous reports [20]. The slides were incubated at 4℃ overnight with primary antibodies recognizing galectin-1 (1:150), collagen I (1:200), collagen III (1:200), and FN1 (1:200), followed by incubation with biotin-conjugated secondary antibodies and HRP-conjugated streptavidin. After 30 min, the sections were stained with diaminobenzidine, counterstained with hematoxylin, and mounted and cover-slipped. The results were assessed by two independent pathologists who had no knowledge of the patients’ clinical status. Galectin-1 was evaluated by the semi-quantitative immunoreactivity score (IRS). The staining intensity was scored as 0 (negative), 1 (weak), 2 (moderate), or 3 (strong), and the percentage of stained cells was scored as 1, 0–5%; 2, 6–25%; 3, 26–50%; or 4, 50–100%. The IRS was obtained by multiplying the staining intensity by the percentage of stained cells. To quantify the collagen I, collagen III, and FN1 immunostaining in the animal model peritoneal tissues, the slides were imaged digitally with equal light exposure, then the average integrated optical density (IOD) was evaluated by Image-Pro Plus (Media Cybernetics, San Diego, CA, USA).

#### Tumor cell adhesion assay

The HPMCs monolayers were cultured in 24-well plates to 80% confluence. Then, the HPMCs were treated with conditioned medium (CM) for 72 h until the HPMCs were completely confluent. The GC cells were stained with 15 μM calcein-AM for 30 min at 37 °C and 5% CO_2_. Then, the stained GC cells (3 × 10^5^/well) were added to the plates containing HPMCs and incubated at 37℃ in 5% CO_2_ for 3 h. The non-adherent tumor cells were removed by washing three times with 1 mL culture medium per well. The remaining adherent tumor cells were observed under a fluorescence microscope, where each well was recorded at 419 nm excitation and 514 nm emission wavelengths by a spectrofluorometer. The average optical density of GC cells adherent to the treated HPMCs was analyzed with Image-Pro Plus.

#### Animal assays

BALB/c athymic nude mice (male, 5 weeks old, 18–21 g) were from the Comparative Medicine Centre of Yangzhou University (Jiangsu, China). The mice were bred in a pathogen-free laminar flow cabinet with a 12-h dark–light cycle at 24 °C and 40–60% relative humidity conditions. The mice were provided with food and purified water and were allowed to adapt to their environment for 1 week before the experiment. Following the principle of minimizing the use of animals, we selected a specific cell line for animal model. To enhance the accuracy of the animal model, we chose cell line SGC-7901 for the animal assays, which demonstrated relatively high differentiation and weak alloplasm adhesion. The mice were divided into three groups (*n* = 6 per group) for the GC peritoneal metastasis model and were intraperitoneally injected with SGC-7901 cells (1.2 × 10^7^) transfected with *LGALS1* overexpression or NC lentiviral vector, or wild-type control (WC) cells. After 56 days, the mice were killed and dissected, the number of peritoneal metastatic nodules were counted, and HE, Masson trichrome staining and IHC evaluation were performed.

#### Masson trichrome staining

Peritoneal tissues from patients with GC and the animal models were paraffin-embedded after formalin fixation and verified with HE staining. Sections (4-μm thick) were stained with Masson trichrome stain and baked at 60℃ for 1 h before dewaxing to water. The sections were immersed in reagent A overnight at room temperature in strict compliance with the manufacturer’s instructions (Solarbio, Beijing, China). The collagen thickness was determined after Masson trichrome staining, where 10 independent measurements were calculated per section and the average was recorded.

### Statistical analysis

Statistical analysis was conducted with SPSS 25 (IBM Corp., Armonk, NY, USA). The continuous variables are expressed as the means ± standard error (SE). Based on normal data distribution, multiple comparisons were performed with one-way analysis of variance and Dunnett’s t-test. Pearson correlation coefficients were used for correlation analysis. *P* < 0.05 and *P* < 0.01 were considered statistically significant.

## Results

### Galectin-1 expression in GC is related to clinical staging and peritoneal fibrosis

**Fig. 1:**
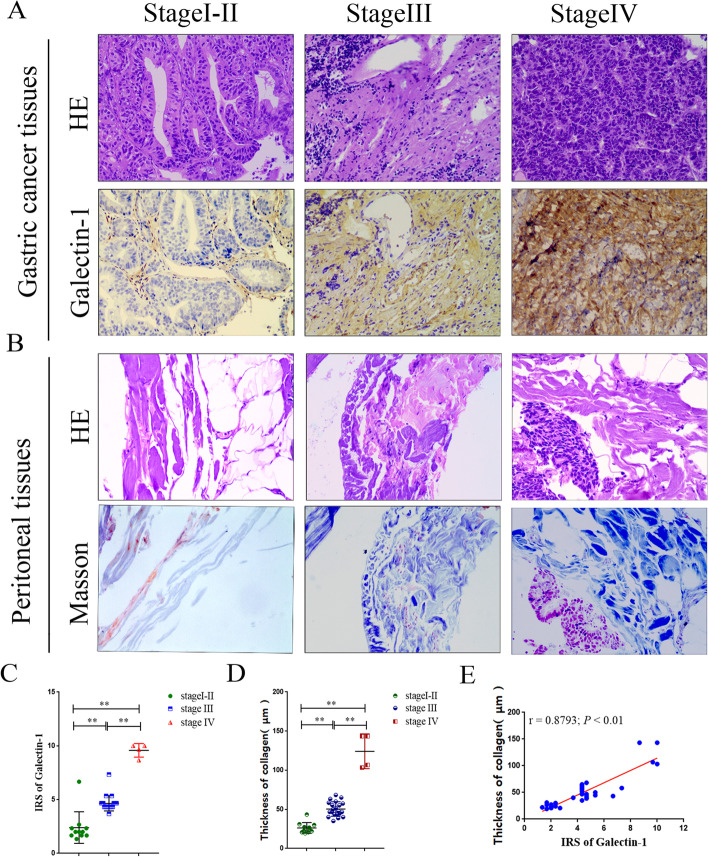
Galectin-1 expression in GC is related to clinical staging and peritoneal fibrosis. **A** Representative images of HE and IHC staining for galectin-1 in GC tissue(× 400 magnification). **B** Representative images of HE and Masson trichrome staining of matched peritoneum tissues (× 400 magnification, GC metastases were observed in the peritoneum at stage IV). **C** Comparison of IRS in different GC stages. **D** Comparison of collagen thickness in different GC stages. **E** The galectin-1 IRS was positively correlated with collagen thickness in GC. GC, Gastric cancer; HE, hematoxylin–eosin; IHC, immunohistochemistry; IRS, immunoreactivity score. **P < 0.01

To confirm the relationship between galectin-1 expression and clinical staging in GC, 36 GC cases were divided into the following groups: stage I–II (*n* = 11), stage III (*n* = 21), and stage IV (*n* = 4) according to the eighth edition AJCC staging system. The clinicopathological features of the patients are described in Table 2. Galectin-1 expression of the primary GC tissues was investigated with IHC staining. The stage IV had a group significantly higher galectin-1 IRS than the other two groups, and the stage III group had a higher galectin-1 IRS than the stage I–II group (all, *P* < 0.01; Fig. 1A, 1C). To explore the relationship between GC clinical staging and collagen deposition in matched peritoneal tissues, collagen deposition in matched peritoneal tissues was detected with HE and Masson trichrome staining. The stage IV group had significantly thicker collagen deposition thickness in the peritoneal tissues than the other two groups (Fig. 1B) while the stage III group had thicker collagen deposition in the peritoneal tissues than the stage I–II group (all, *P* < 0.01; Fig. 1D). Galectin-1 expression in the primary GC tissues correlated significantly with collagen deposition thickness in the matched peritoneal tissues (*r* = 0.8793, *P* < 0.001; Fig. 1E).

**Table 2:** Clinicopathological features in patients with GC

All patients	I-II stage	III stage	IV stage	*P*
	11(%)	21(%)	4(%)	
Sex				0.172
Male	8 (72.7)	15 (71.4)	1 (25.0)	
Female	3 (27.3)	6 (28.6)	3 (75.0)	
Age (years)				0.636
> 65	9 (81.8)	17(81.0)	4 (100)	
≤ 65	2 (18.2)	4(19.0)	0(0)	
Tumor diameter (cm)				0.343
> 5	4 (36.4)	12 (57.1)	3 (75.0)	
≤ 5	7 (63.6)	9 (42.9)	1 (25.0)	
Pathological classification				0.03
I–II	5(45.5)	2(9.5)	0	
III	6 (54.5)	19(90.5)	4(100.0)	
Depth of invasion				< 0.001
T1	6 (54.5)	0	0	
T2–T3	5(45.5)	2(9.5)	0	
T4	0	19(90.5)	4(100.0)	
Lymph node metastasis				< 0.001
N0	9 (81.8)	2(9.5)	0	
N1	1(9.1)	2(9.5)	0	
N2	1(9.1)	7 (33.3)	0	
N3	0	10(47.7)	4(100.0)	
Tumor emboli in the micro-vessels				0.008
Negative	6 (54.5)	2(9.5)	0	
Positive	5(45.5)	19(90.5)	4(100.0)	
Nerve invasion				< 0.001
Negative	8 (72.7)	2(9.5)	0	
Positive	3 (27.3)	19(90.5)	4(100.0)

### *LGALS1* promotes collagen expression in peritoneal mesothelial cells

**Fig. 2:**
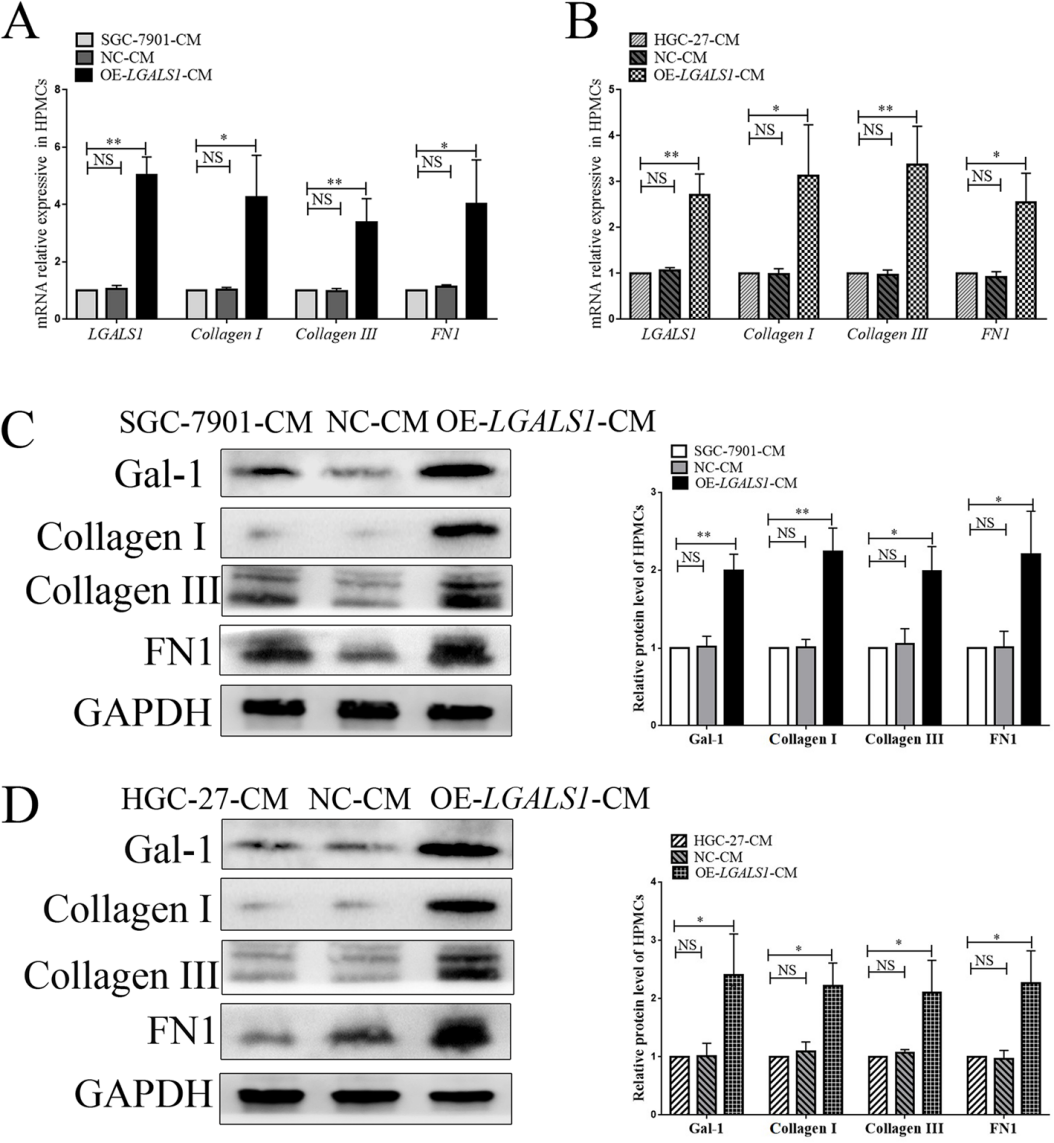
*LGALS1* promotes collagen expression in peritoneal mesothelial cells. **A**, **B** qRT-PCR analysis of *LGALS1*, *COL1A1*, *COL3A1*, and *FN1* mRNA expression in HPMCs treated with (**A**) OE-*LGALS1*-SGC-7901-CM and (**B**) OE-*LGALS1*-HGC-27-CM. **C**, **D** Western blot confirmation of galectin-1, collagen I, collagen III, and FN1 in HPMCs treated with (**C**) OE-*LGALS1*-SGC-7901-CM and (**D**) OE-*LGALS1*-HGC-27-CM. CM, Conditioned medium; Gal-1, galectin-1; HPMCs, human peritoneal mesothelial cells; NC-CM, CM from NC-transfected GC cells; OE-*LGALS1*-CM, CM from *LGALS1* overexpression GC cells; NS, not significant; qRT-PCR, quantitative reverse transcription PCR. *P < 0.05; **P < 0.01

We verified whether *LGALS1* affected the production of ECM (such as collagen I, collagen III, and FN1]) in HPMCs in vitro. We established the following *LGALS1* overexpression (OE-*LGALS1*) GC cell lines via lentivirus transfection: HGC-27 OE-*LGALS1*, SGC-7901 OE-*LGALS1*, NC-transfected (NC), and untransfected WC. The HPMCs were treated with SGC-7901 CM for 72 h before the experiment. Then, the HPMCs were divided into SGC-7901-CM, NC-CM, and OE-*LGALS1*-CM groups. The qRT-PCR results demonstrated significantly high expression of *LGALS1* mRNA in the OE-*LGALS1*-CM-treated HPMCs (*P* < 0.01; Fig. 2A), which demonstrated significantly higher *COL1A1*, *COL3A1*, and *FN1* mRNA levels than the SGC-7901-CM- and NC-CM-treated HPMCs (all,* P* < 0.01; Fig. 2A). *LGALS1* upregulated *COL1A1*, *COL3A1*, and *FN1* mRNA expression in the HPMCs. The protein data were confirmed with western blotting, where the OE-*LGALS1*-CM-treated HPMCs exhibited higher Collagen I, Collagen III, and FN1 proteins expression after 72-h treatment (*P* < 0.01; Fig. 2C). The above experiments were repeated with HGC-27 cells, and the qRT-PCR (Fig. 2B) and western blotting (Fig. 2D) results were consistent with the SGC-7901 cell experiment results.

### *LGALS1* enhances GC cell adhesion to HPMCs

**Fig. 3:**
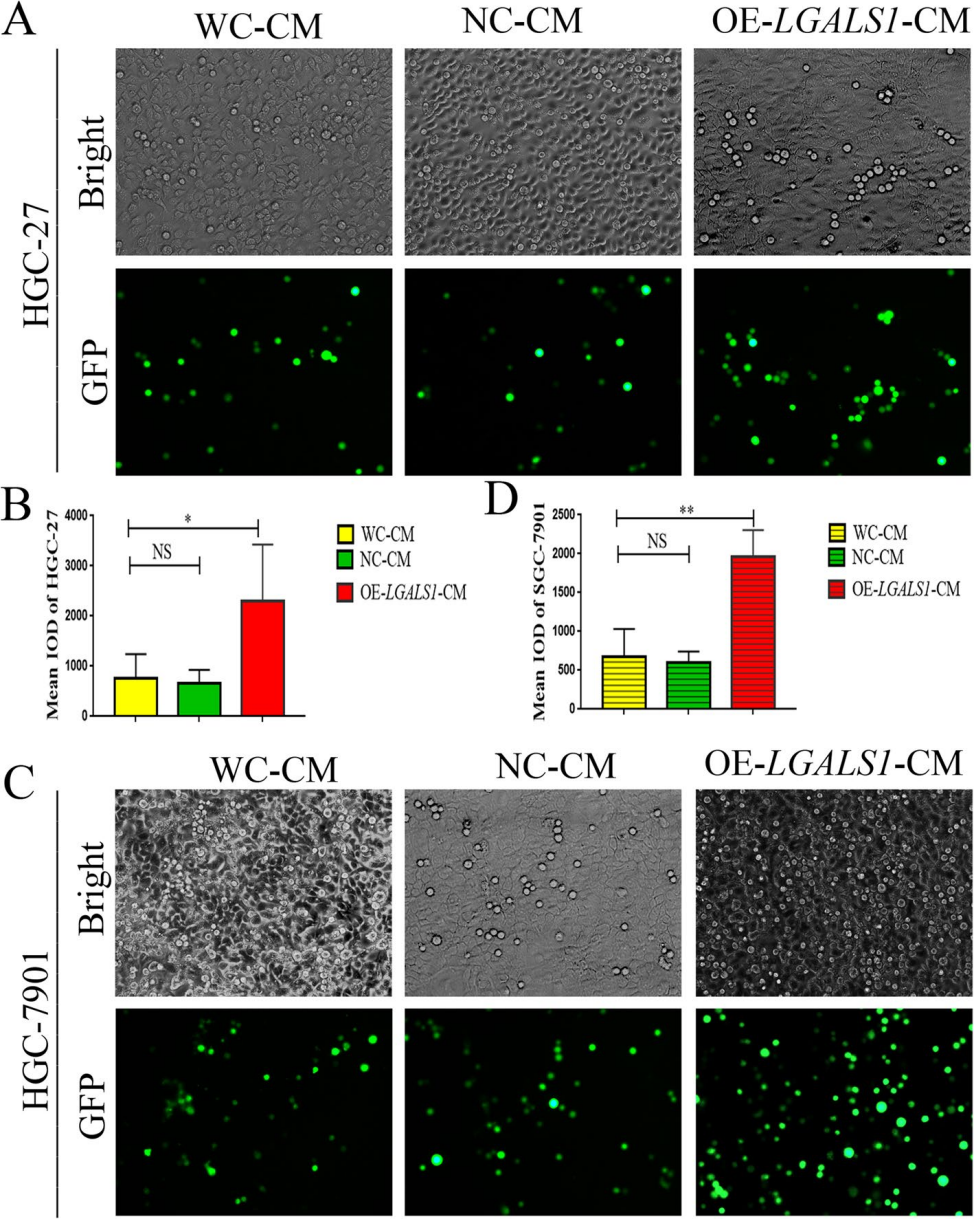
Effects of *LGALS1* on GC cells adhesion to peritoneal mesothelial cells. **A**, **C** HPMCs were incubated with (**A**) OE-*LGALS1*-HGC-27-CM and (**C**) OE-*LGALS1*-SGC-7901-CM for 72 h, then GC cells treated with calcein-AM for 3 h were added to the HPMCs. Top: Bright-field microscopy (× 40 magnification); bottom: fluorescence microscopy (× 40 magnification). (B, D) Mean IOD of (**B**) HGC-27 (**D**) SGC-7901 cells adherent to HPMCs. CM, Conditioned medium; GC, gastric cancer; GFP, green fluorescent protein; HPMCs, human peritoneal mesothelial cells; IOD, integrated optical density; WC-MC, CM from wild-type control cells, NC-CM, CM from negative control-transfected GC cells; OE-*LGALS1*-CM, CM from *LGALS1* overexpression GC cells; NS, not significant. *P < 0.05; **P < 0.01

We performed a cell adhesion assay to verify whether *LGALS1* promoted GC cell adhesion to HPMCs. Fluorescence examination revealed that the IOD of HGC-27 cells adhering to OE-*LGALS1*-HGC-27 CM-treated HPMCs was 2303.10 ± 919.11, which was higher than the IOD of the NC-HGC-27 CM-treated HPMCs (659.53 ± 219.32) and WC-HGC-27 CM-treated HPMCs (764.76 ± 390.20) (*P* < 0.05, Fig. 3A, B). We repeated the adhesion experiment on HPMCs with SGC-7901 CM, where the IOD of SGC-7901 cells adhering to OE-*LGALS1*-CM-treated HPMCs was 1958.20 ± 283.53, which was higher than the IOD of the WC-CM-treated HPMCs (675.97 ± 291.57) and NC-CM-treated HPMCs (602.80 ± 113.97) (*P* < 0.01, Fig. 3C, D). This result demonstrated that *LGALS1* appeared to promote GC cell adherence to HPMCs.

### *LGALS1* promotes GC peritoneal metastasis through peritoneal fibrosis

**Fig. 4:**
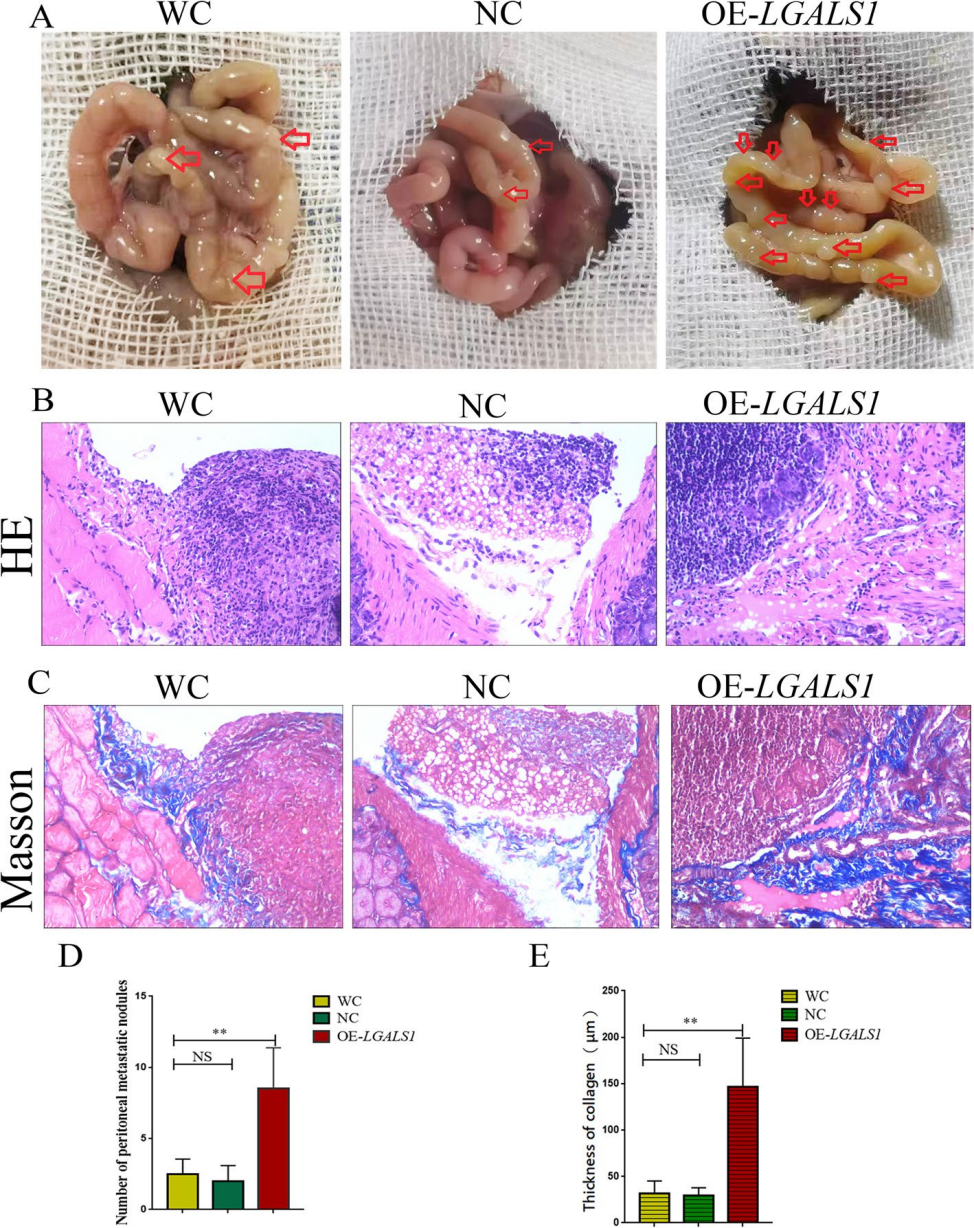
*LGALS1* promotes peritoneal metastasis of GC through peritoneal fibrosis. **A** Representative images of animal models of peritoneal metastasis. Red arrows indicate metastatic cancer nodules. **B** HE staining of cancer nodules on the peritoneal surface (× 400 magnification). **C** Masson trichrome staining of basal collagen deposition at the cancer nodule(× 400 magnification). **D** The number of peritoneal metastatic nodules and (E)collagen thickness are expressed as the mean ± SE. GC, Gastric cancer; HE, hematoxylin–eosin; WC, wild-type control GC cells; NC, negative control-transfected GC cells; OE-*LGALS1*, *LGALS1* overexpressing GC cells; NS, not significant; SE, standard error. **P < 0.01

Based on the results above, we designed in vivo experiments in animal models to elucidate the relationship between *LGALS1* and GC peritoneal metastasis. The results demonstrated that when tumor cells were injected intraperitoneally to simulate tumor cell shedding and peritoneal metastasis, the peritoneal metastases nodules were not statistically significantly different between the WC and NC groups, while the OE-*LGALS1* group exhibited significantly more peritoneal metastases nodules than the control groups (*P* < 0.01, Fig. 4A, D). HE staining demonstrated cancer nodules on the peritoneal surface (Fig. 4B) and Masson trichrome staining demonstrated basal collagen deposition in the cancer nodules (Fig. 4C). The basal collagen deposition thickness of the peritoneal metastases nodules was 31.33 ± 12.63 μm in the WC group and 29.00 ± 8.08 μm in the NC group, which was not statistically significantly different. The peritoneal metastases nodule basal collagen thickness in the OE-*LGALS1* group was 146.67 ± 47.84 μm, which was significantly thicker than that in the control group (*P* < 0.01, Fig. 4E).

### *LGALS1* promotes GC peritoneal metastasis by enhancing peritoneal collagen deposition

**Fig. 5:**
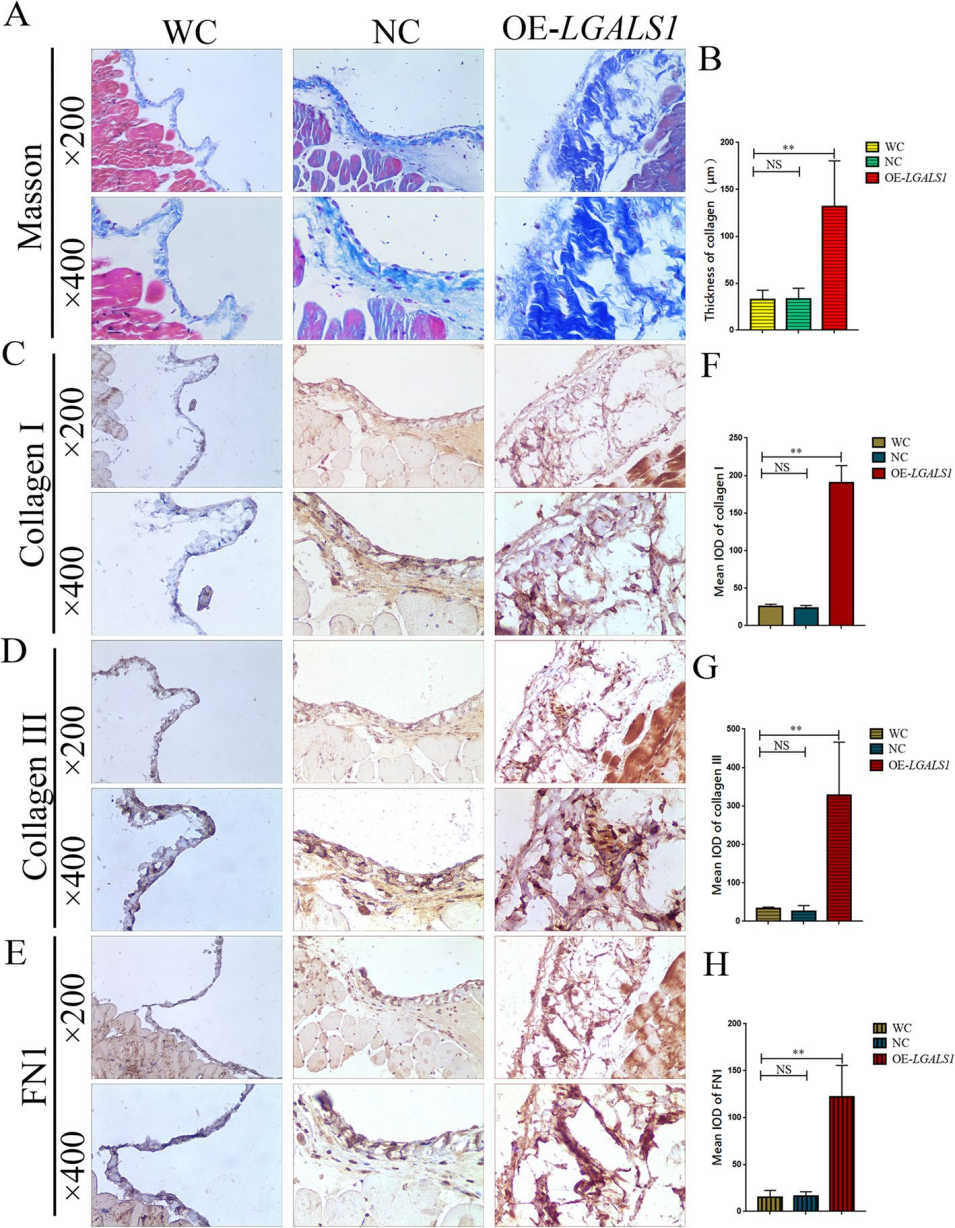
*LGALS1* promotes peritoneal metastasis of GC by enhancing peritoneal collagen deposition. **A** Masson trichrome staining of peritoneal collagen deposition in the animal model. **B** The collagen thickness is expressed as the mean ± SE. **C**–**E** IHC of (**C**) collagen I, (**D**) collagen III, and (**E**) FN1 in the animal model peritoneum. **F**–**H** The IOD (mean ± SE) of (**F**) collagen I, (**G**) collagen III, and (**H**) FN1. FN1, Fibronectin 1; GC, gastric cancer; IOD, integrated optical density; IHC, immunohistochemistry; WC, wild-type control GC cells; NC, negative control-transfected GC cells; OE-*LGALS1*, *LGALS1* overexpression GC cells; NS, not significant; SE, standard error. *P < 0.05; **P < 0.01

To clarify whether *LGALS1* promoted GC peritoneal metastasis by enhancing peritoneal collagen deposition, Masson trichrome staining and IHC were used to observe collagen deposition and collagen expression in nude mouse peritoneal tissues. Our results demonstrated that the collagen deposition thickness at the peritoneal tissues in the OE-*LGALS1* group was 131.67 ± 44.50 μm, which was significantly thicker than that in the WC group (32.33 ± 9.29 μm) and NC group (32.33 ± 10.83 μm) (*P* < 0.01, Fig. 5A, B). The IHC results demonstrated that the mean IOD of collagen I in the OE-*LGALS1* group was higher than in the WC and NC groups (*P* < 0.01, Fig. 5C, F). The IHC of collagen III and FN1 yielded similar results, where the mean IOD of collagen III (*P* < 0.01, Fig. 5D, G) and FN1 (*P* < 0.01, Fig. 5E, H) in the OE-*LGALS1* group were also higher than that of the control groups. Our results confirmed that *LGALS1* promoted GC peritoneal metastases formation in the animal models by promoting peritoneal collagen deposition.

## Discussion

Peritoneal metastasis is the most common and complicated-to-treat postoperative metastasis in patients with GC [21]. However, the molecular mechanism regulating peritoneal metastasis in GC remains unclear. Revealing the molecular mechanism of GC peritoneal metastasis and preventing peritoneal metastasis is clinically more important than treating it. Previously, we reported that galectin-1 regulates malignant biological behaviors such as GC invasion and migration [20], and speculated that galectin-1 expression in GC tissues might be related to GC peritoneal metastasis [19].

In this study, we used IHC to detect galectin-1 protein in 36 patients with GC, where the galectin-1 IRS revealed that the galectin-1 expression level in GC tissues was closely related to the clinical stage, which was consistent with the previous study results [17]. We also determined that collagen deposition in the peritoneal tissues of patients with GC was closely related to clinical staging, which was also consistent with previous findings [22]. The presence of collagen in the GC microenvironment and stromal fibrosis affects GC metastasis [23, 24]. The most recent analysis of the mRNA expression of collagen family genes (*COL10A1* and *COL11A1*) in different T stages of GC determined that *COL10A1* and *COL11A1* mRNA expression was significantly upregulated in the late T stage [25]. Therefore, collagen is involved in GC progression and metastasis. Notably, we determined that galectin-1 expression in GC tissues was positively correlated with the collagen thickness in the matched peritoneal tissues. Therefore, we hypothesized that galectin-1 in GC tissues promotes peritoneal metastasis by promoting peritoneal collagen deposition.In vitro, we treated HPMCs with CM from OE-*LGALS1* GC cell lines,and we observed a significantly high expression of *LGALS1* mRNA in the OE-*LGALS1*-CM-treated HPMCs. As we know, galectin-1 is synthesized on cytosolic ribosomes, translocated to the intracellular side of cell membranes and then secreted to the extracellular [19]. Therefore, we inferred that there are more exogenous galectin-1 in OE-*LGALS1*-CM, and extracellular galectin-1 can activate multiple intracellular signaling pathways through the transmembrane receptor integrin-β1 on cell membrane, such as PI3K/AKT [19, 26]. The PI3K/Akt/mTOR signaling pathway plays a crucial role in regulating HIF-1 activation [27]. Moreover, HIF-1 induces increased galectin-1 expression by binding to hypoxia-responsive elements in the *LGALS1* promoter region [28], forming a vicious cycle of "hypoxia-galectin-1—sustained hypoxia". However, these are merely theoretical inferences. In this study, we only made preliminary observations, and the specific mechanisms will be further explored in subsequent studies, including the expression of various cytokines in conditioned medium and differences in protein expression in peritoneal mesothelial cells after conditioned medium treatment.

In vitro, we determined that *LGALS1* promoted high *COL1A1*, *COL3A1*, and *FN1* mRNA expression in the HPMCs and promoted high collagen I, collagen III, and FN1 protein expression. We conducted cell adhesion experiments to explore how HPMCs with high collagen expression are more prone to peritoneal metastasis, where the OE-*LGALS1*-CM-treated HPMCs had stronger adhesion ability to GC cells. Collagen deposition and adhesion processes that promote tumor progression and metastasis have been demonstrated in pancreatic cancer [29] and lung adenocarcinoma [30]; our results indicated that the increased collagen expression in the HPMCs promoted GC cell adhesion and enhanced the occurrence of GC peritoneal metastasis.

In vivo, overexpressing *LGALS1* in GC cells promoted peritoneal metastatic nodule formation in nude mice, and Masson trichrome staining demonstrated that *LGALS1* overexpression significantly increased basal collagen deposition at the metastatic nodule. Increased matrix protein deposition promotes tumor progression by interfering with cell polarity and cell–cell adhesion and ultimately amplifies growth factor signaling pathways [31].

Surprisingly, IHC demonstrated that *LGALS1* overexpression in GC cells significantly promoted collagen I, collagen III, and FN1 protein expression in the nude mouse peritoneal tissue from which metastases was absent. Therefore, we believe that *LGALS1* might reshape the peritoneal TME before peritoneal metastasis and establish a premetastasis niche.

An important ECM component, collagen substantially influences cancer pathogenesis and progression [32], which determines the functional properties of the matrix. Changes in collagen deposition or degradation can lead to a decline in ECM homeostasis. Increased collagen crosslinking and deposition contributed to tumor progression through increased integrin signaling [33, 34]. GC peritoneal dissemination is characterized by abundant collagen deposition in the peritoneum, and changes in serum collagen IV can be detected in patients with GC at the early stage of peritoneal metastasis [35]. Collagen I is composed of collagen type I alpha 1 (*COL1A1*) and *COL1A2*. *COL1A1* and *COL1A2* mRNA expression in GC tissues is significantly higher than that in normal gastric mucosa tissues, and higher *COL1A1* and *COL1A2* expression levels were associated with lower overall survival rates [36].* COL1A1 *may be a potential monitoring factor for screening early GC, and *COL1A1 *and *COL1A2* may predict poor clinical prognosis in patients with GC [36]. As *COL3A1 *is also highly expressed in GC cells, the collagen family genes might serve as GC progression and prognosis markers [31].

FN1 is involved in tumor occurrence and development and is upregulated in various cancers, such as head and neck squamous cell carcinoma [37] and cadmium-related bladder cancer [38]. Encoding fibronectin, *FN1* is a pivotal signaling gene for therapeutic intervention against pancreatic cancer [39]. FN1 is upregulated in GC tissues compared with neighboring normal tissues, rendering it a potential biomarker for poor prognosis in patients with GC [40].

In this study, we determined that *LGALS1* promoted *COL1A1*, *COL3A1*, and *FN1* mRNA and protein expression in HPMCs; promoted collagen I, collagen III, and FN1 protein expression in animal model peritoneal tissues; and promoted peritoneal metastases formation in the animal model. No studies have reported that galectin-1/*LGALS1* directly promotes collagen expression, but galectin-1 indirectly regulates collagen expression through the galectin-1–TGF-β–EMT (epithelial–mesenchymal transition) pathway in many diseases, such as diabetic retinopathy [41], chronic pancreatitis/pancreatic cancer [42], and liver fibrosis [43]. Previously, we reported that galectin-1/*LGALS1* activated the TGF-β1–SMAD2/3 signaling pathway and promoted EMT in GC cells. TGF-β-mediated EMT-like changes in HPMCs through Smad2 phosphorylation promotes gastric cancer growth and fibrosis [44].Therefore, we speculated that galectin-1/*LGALS1* promoted the expression of the cytokine TGF-β1 in GC tissues, and TGF-β1 was released into the blood or ascites to activate the TGF-β1–SMAD2/3 signaling pathway and promote EMT in peritoneal mesenchymal cells, thereby promoting peritoneal collagen deposition. However, this hypothesis should be examined experimentally.

In summary, as a novel promoter of GC peritoneal metastasis, galectin-1/*LGALS1* promoted peritoneal metastasis formation by inducing collagen deposition in peritoneal tissue and increasing peritoneal adhesion to GC cells. Finally, we identified galectin-1/*LGALS1* as a potential molecule for GC peritoneal metastasis.

## Supplementary Information

**Additional file 1.**

## Data Availability

All the data used to support the findings of this study are included within the article.

## Abbreviations

AJCC: American Joint Committee on Cancer
ARRIVE: Animal Research: Reporting of In Vivo Experiment
CAFs: Cancer-associated fibroblasts
CM: Conditioned medium
COL1A1: Collagen I
COL3A1: Collagen III
ECM: Extracellular matrix
EMT: Epithelial–mesenchymal transition
FBS: Fetal bovine serum
FN1: Fibronectin 1
GAPDH: Glyceraldehyde-3-phosphate dehydrogenase
GC: Gastric cancer
GFP: Green fluorescent protein
HE: Hematoxylin–eosin
HPMC: Human peritoneal mesothelial cell
HRP: Horseradish peroxidase
IHC: Immunohistochemistry
IOD: Integrated optical density
IRS: Immunoreactivity score
NC: Negative control
OE: Overexpression
qRT-PCR: Quantitative reverse transcription PCR
SE: Standard error
TME: Tumor microenvironment
TNM: Tumor-node-metastasis
WC: Wild-type control
