# Supplementary material for: Galectin-1 promotes gastric cancer peritoneal metastasis through peritoneal fibrosis
Source: BMC Cancer. 2023 Jun 17;23:559. doi: 10.1186/s12885-023-11047-2 (PMC10276408; doi:10.1186/s12885-023-11047-2)

Galectin-1 for Figure. 2

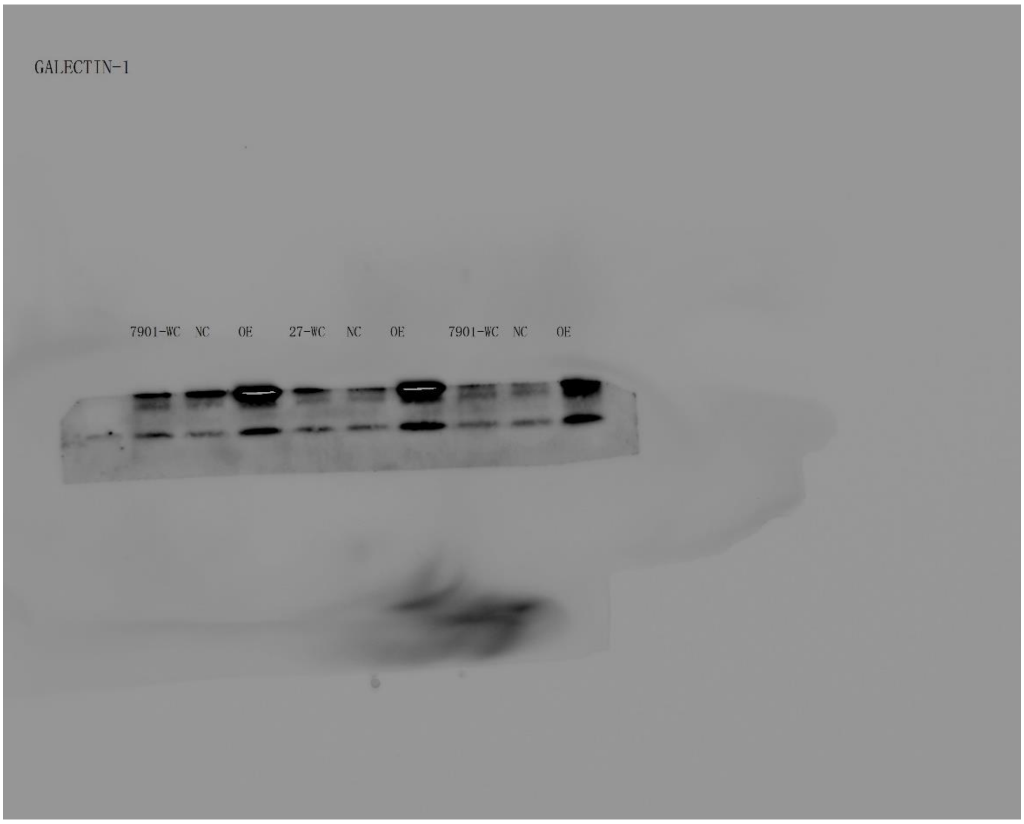

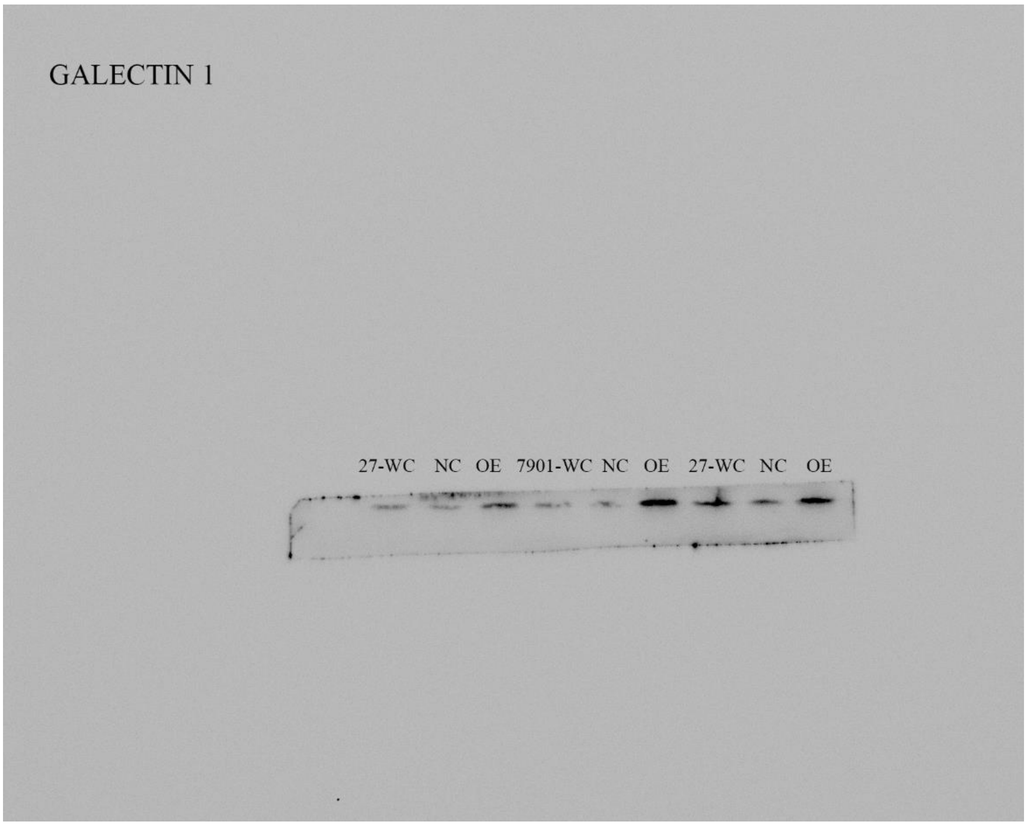

Collagen I for Figure. 2

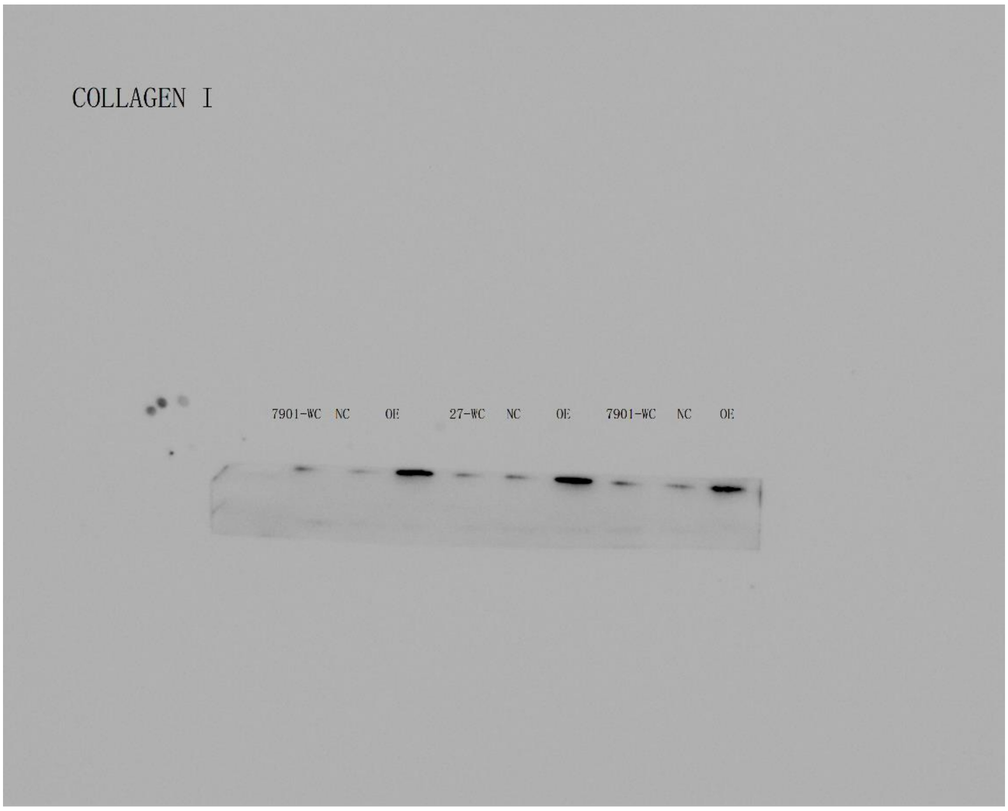

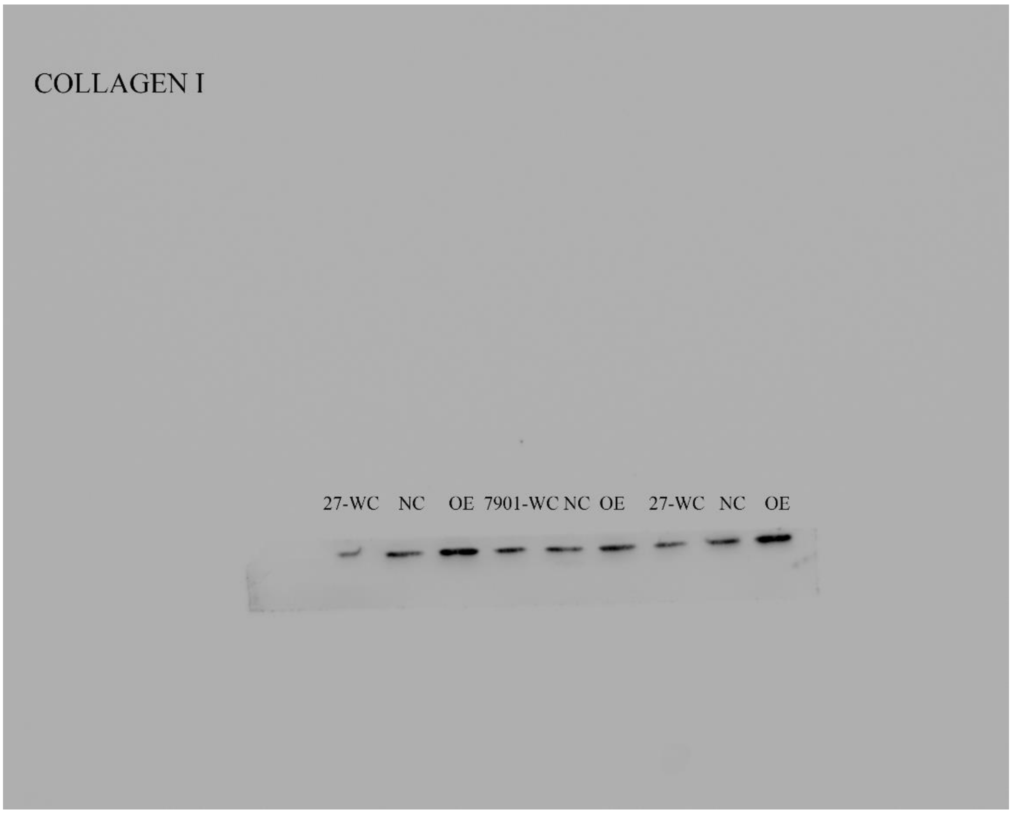

Collagen III for Figure. 2

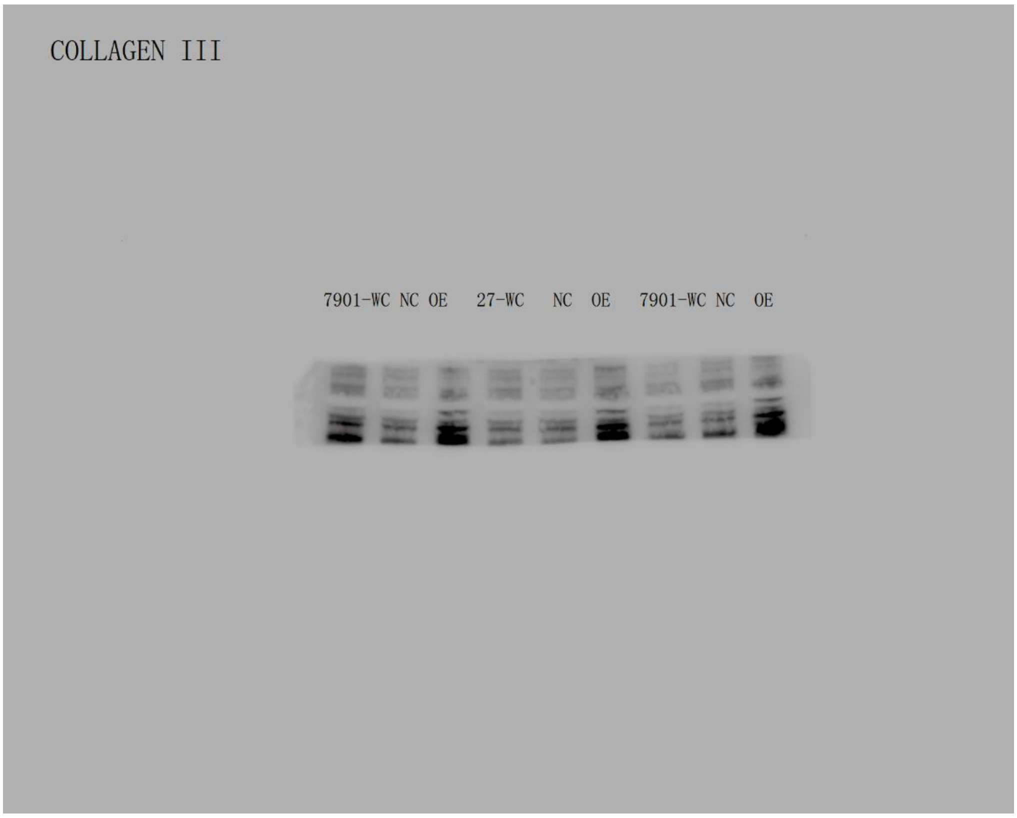

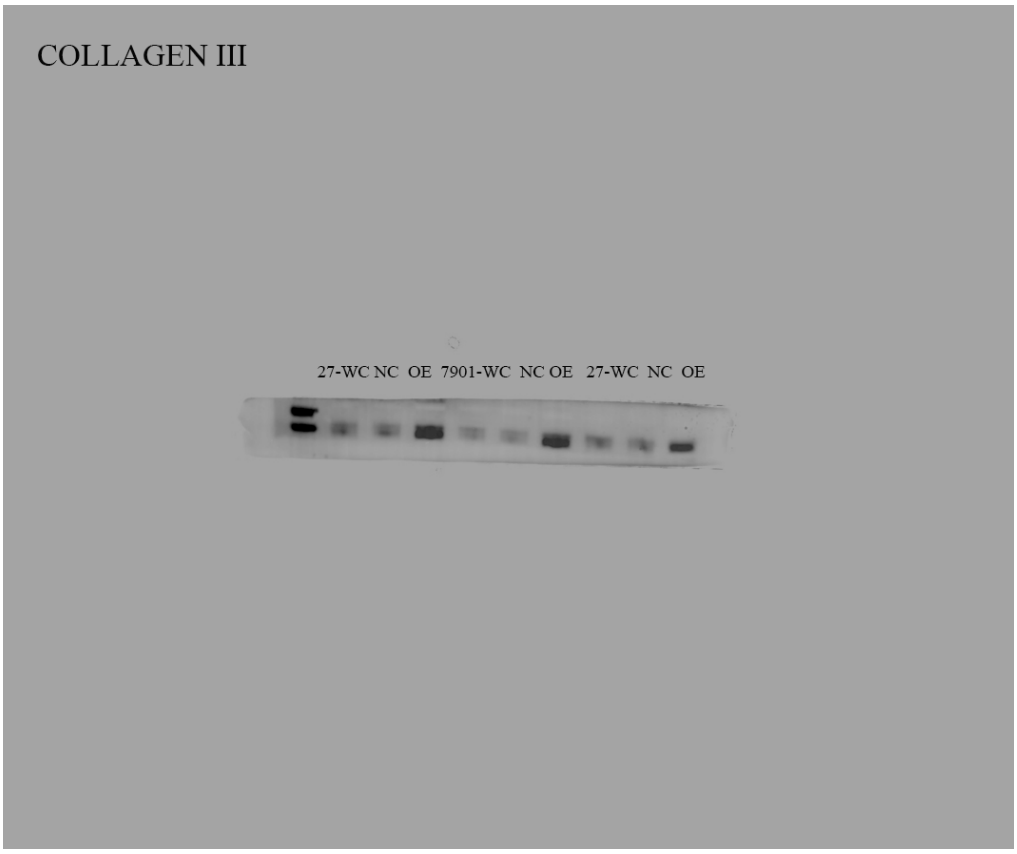

FN1 for Figure. 2

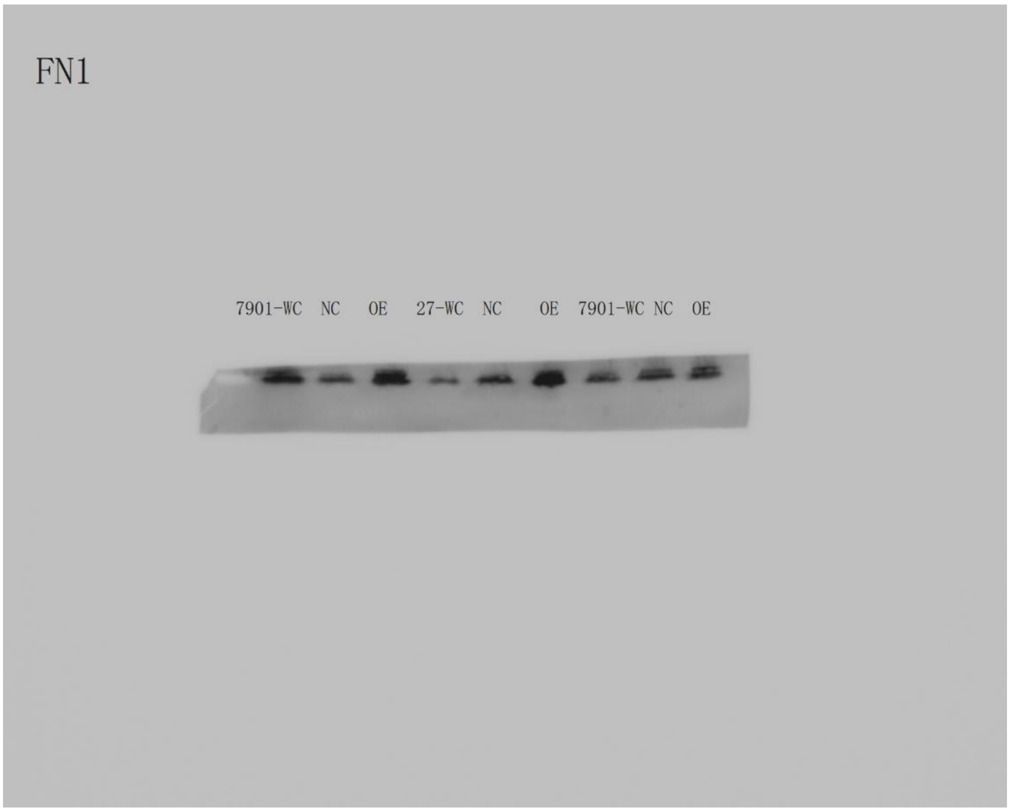

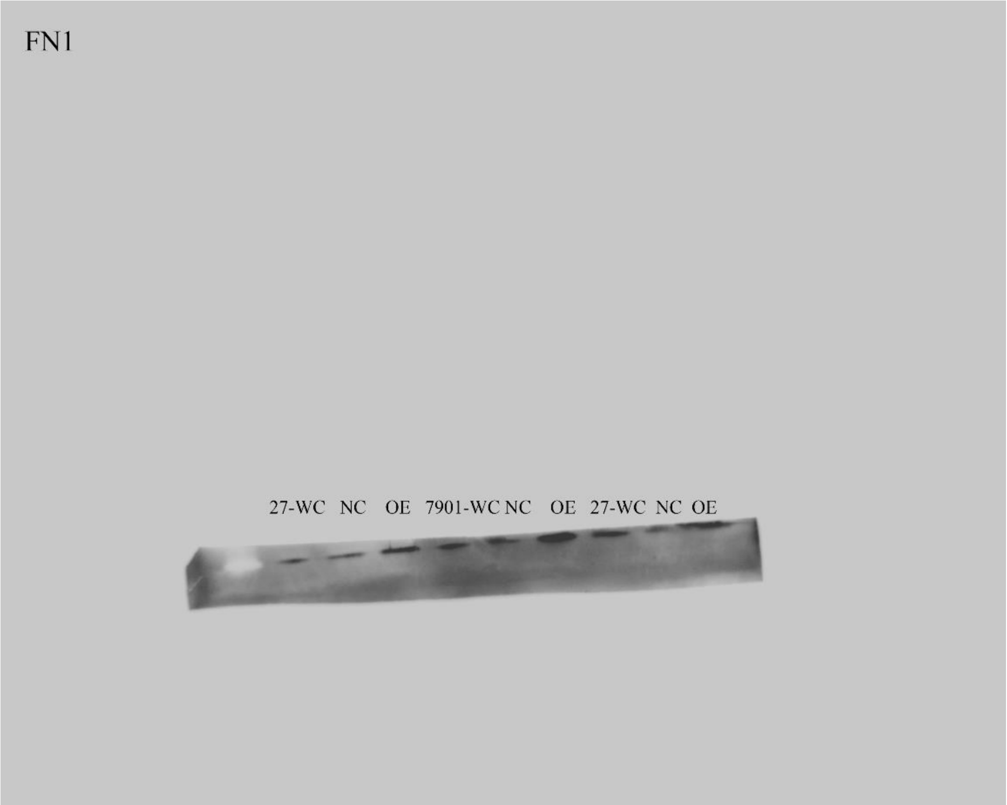

GAPDH for Figure. 2

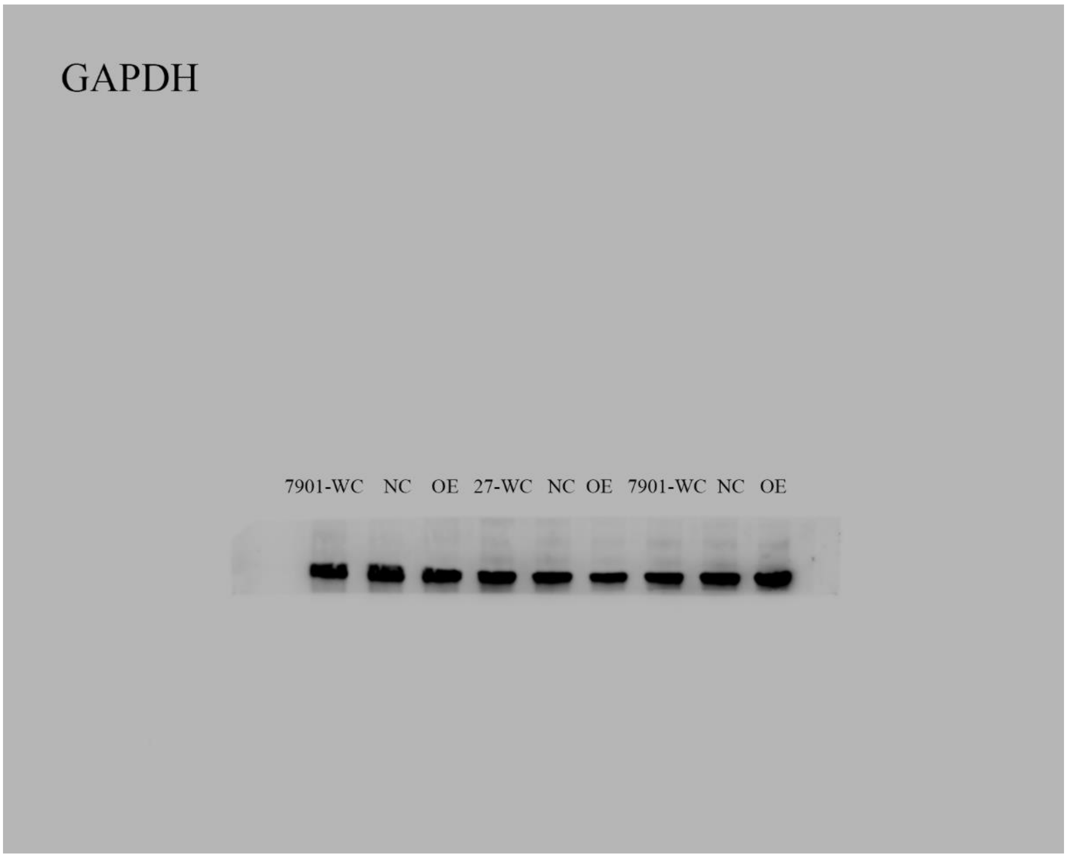

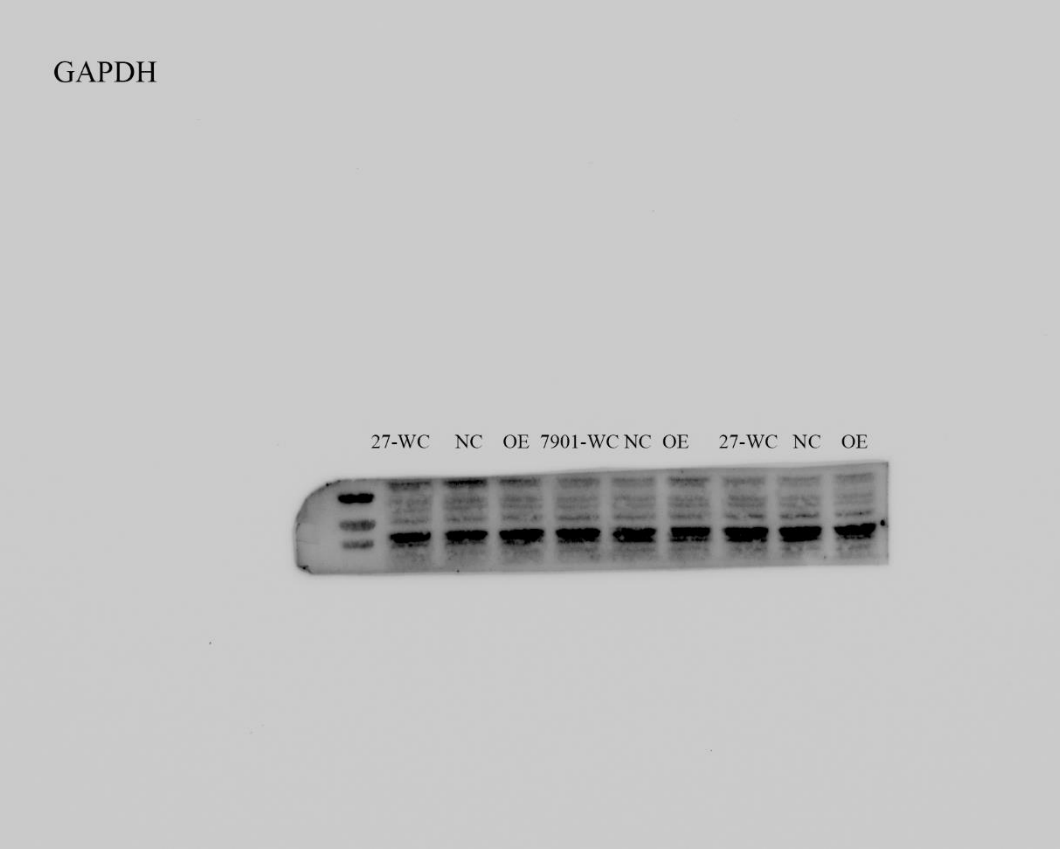

Supplement: Supplementary file 1 — Additional file 1. [file 12885_2023_11047_MOESM1_ESM.pdf]
